# Single-cell RNA sequencing reveals a unique pericyte type associated with capillary dysfunction

**DOI:** 10.7150/thno.83532

**Published:** 2023-04-23

**Authors:** Min Xia, Lyu Jiao, Xiao-Han Wang, Min Tong, Mu-Di Yao, Xiu-Miao Li, Jin Yao, Dan Li, Pei-Quan Zhao, Biao Yan

**Affiliations:** 1 The Fourth School of Clinical Medicine, Nanjing Medical University, Nanjing 210000, China.; 2 The Affiliated Eye Hospital, Nanjing Medical University, Nanjing 210000, China.; 3 Department of Ophthalmology, Xinhua Hospital Affiliated to Shanghai Jiaotong University School of Medicine, Shanghai 200092, China.; 4 Eye Institute, Eye & ENT Hospital, Shanghai Medical College, Fudan University, Shanghai 200030, China.; 5 NHC Key Laboratory of Myopia (Fudan University), Key Laboratory of Myopia, Chinese Academy of Medical Sciences, and Shanghai Key Laboratory of Visual Impairment and Restoration (Fudan University), Shanghai 200030, China.

**Keywords:** Pericyte, Single-cell RNA sequencing, Capillary dysfunction, Pericyte-myofibroblast transition

## Abstract

**Background:** Capillary dysfunction has been implicated in a series of life- threatening vascular diseases characterized by pericyte and endothelial cell (EC) degeneration. However, the molecular profiles that govern the heterogeneity of pericytes have not been fully elucidated.

**Methods:** Single-cell RNA sequencing was conducted on oxygen-induced proliferative retinopathy (OIR) model. Bioinformatics analysis was conducted to identify specific pericytes involved in capillary dysfunction. qRT-PCRs and western blots were conducted to detect Col1a1 expression pattern during capillary dysfunction. Matrigel co-culture assays, PI staining, and JC-1 staining was conducted to determine the role of Col1a1 in pericyte biology. IB4 and NG2 staining was conducted to determine the role of Col1a1 in capillary dysfunction.

**Results:** We constructed an atlas of > 76,000 single-cell transcriptomes from 4 mouse retinas, which could be annotated to 10 distinct retinal cell types. Using the sub-clustering analysis, we further characterized retinal pericytes into 3 different subpopulations. Notably, GO and KEGG pathway analysis demonstrated that pericyte sub-population 2 was identified to be vulnerable to retinal capillary dysfunction. Based on the single-cell sequencing results, Col1a1 was identified as a marker gene of pericyte sub-population 2 and a promising therapeutic target for capillary dysfunction. Col1a1 was abundantly expressed in pericytes and its expression was obviously upregulated in OIR retinas. Col1a1 silencing could retard the recruitment of pericytes toward endothelial cells and aggravated hypoxia-induced pericyte apoptosis *in vitro*. Col1a1 silencing could reduce the size of neovascular area and avascular area in OIR retinas and suppressed pericyte-myofibroblast transition and endothelial-mesenchymal transition. Moreover, Col1a1 expression was up-regulated in the aqueous humor of the patients with proliferative diabetic retinopathy (PDR) or retinopathy of prematurity (ROP) and up-regulated in the proliferative membranes of PDR patients.

**Conclusions:** These findings enhance the understanding of the complexity and heterogeneity of retinal cells and have important implications for future treatment of capillary dysfunction.

## Introduction

Capillary dysfunction has been implicated in the pathogenesis of several human diseases, such as cerebral small vessel disease, Alzheimer's, and ocular diseases 1, 2. Capillaries are mainly composed of endothelial cells (ECs) and pericytes. The literatures on the biology of ECs are appreciable but less is known about the biology of pericytes 3. Pericytes are embedded within the basement membrane of capillaries and make direct contacts with ECs. Increasing studies have revealed that pericytes play important roles in capillary development, stabilization, and maturation, which can regulate capillary remodeling 4.

In general, pericytes are morphologically, biochemically, and physiologically heterogeneous 5. Some pericytes can regulate endothelial proliferation, differentiation, and permeability 6. Some pericytes can act as the constituents of basement membrane and extracellular matrix or act as the progenitor cells for vascular repair 7. In addition, some pericytes can synthesize the vasoactive auto-regulating agonists 8. However, the molecular profile and the heterogeneity of pericytes is still not fully understood.

Determination of gene profiles in different types of pericytes may provide important insights into pericyte heterogeneity. Recently, the advances in single-cell RNA sequencing have allowed for comprehensive analysis of cellular and molecular information in a specific tissue 9. Single-cell RNA sequencing can be used to investigate cell-to-cell transcriptomic variation, identify novel or rare cell types, and provide novel insights into physiological and pathological processes 10. However, the profile of pericyte heterogeneity and the implications of pericyte subpopulations during capillary dysfunction has not been fully understood.

Oxygen-induced retinopathy mouse model (OIR) is a well-established tool for studying capillary dysfunction. In this model, the neonatal mice were exposed to 75% oxygen from postnatal (P) day 7 to P12, followed by the exposure to room air (RA) from P12 to P17. P7-P12 defines phase 1 of OIR, during which immature vessels regress. The transfer of neonatal mice to room air at P12 results in relative hypoxia in the vaso-obliteration regions that stimulates pro-angiogenic factors. P12-P17 defines phase 2 of OIR, which is characterized by pathological angiogenesis at the junction of avascular and vascularized retina 11. In this study, we combined single-cell RNA sequencing with immunofluorescence staining, *in vitro* studies, and *in vivo* studies to comprehensively profile pericyte subpopulations at phase 2 of OIR model. We identified 3 different subpopulations of pericytes during pathological angiogenesis. Pericyte sub-population 2 was found to be vulnerable to pathological angiogenesis, which was characterized by abundant expression of Col1a1. Col1a1 silencing could suppress pathological angiogenesis via affecting pericyte degeneration. Taken together, this study provides novel insights into the transcriptomic complexity and heterogeneity alterations of retinal cell types and sheds light on the treatment of capillary dysfunction.

## Results

### Single-cell profiles of retinas isolated from OIR mice and room air (RA) control mice

**Figure 1:**
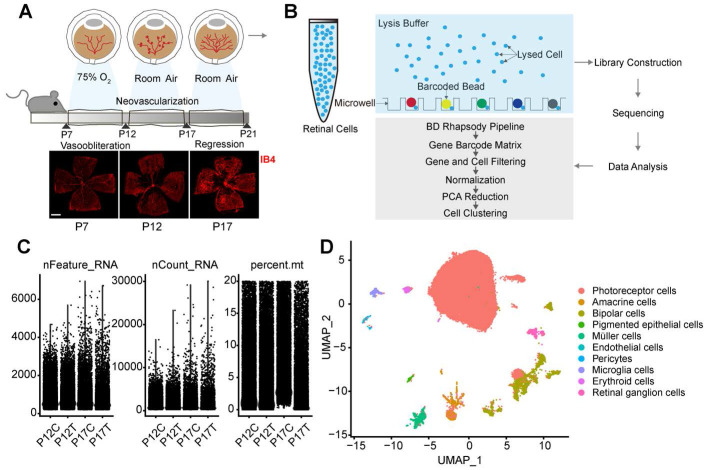
** Single-cell profile of retinas isolated from OIR mice and room air (RA) control mice.** (A) The neonatal mice were exposed to 75% oxygen from postnatal (P) day 7 to P12, followed by the exposure to room air (RA) from P12 to P17 to build OIR model. Retinal vessels were visualized by staining with Isolectin IB-4 for 1 h at room temperature. Scale bar: 500 μm. (B) The schematic overview of the experimental protocol. The retinas were collected from OIR mice (P12T and P17T) and RA control mice (P12C and P17C) and enzymatically dissociated for 1 h at 37ºC. The single-cell suspension was sequenced using the droplet-based massively parallel scRNA-sequencing. (C) Violin plot showing gene number in each cell (nFeature), total count number of all genes in each cell (nCount), and percentage of mitochondrial gene number in total gene number in each cell (percent.mt). (D) Uniform Manifold Approximation and Projection (UMAP) representation of single-cell gene expression showing 10 identified retinal cell types. Single cells were color-coded by cluster annotation.

To investigate the adaptive responses and structure changes of retinal vessels during pathological angiogenesis, we established a murine model of OIR and collected the retinas from OIR mice (P12T and P17T) and RA control mice at P12 and P17 (P12C and P17C) and subjected to single-cell RNA sequencing (Figure 1A-B). After obtaining the single-cell RNA sequencing data, quality filtering and batch correction were carried out. Three indicators were used for data analysis, including gene number in each cell (nFeature), total count number of all genes in each cell (nCount), and percentage of mitochondrial gene number in total gene number in each cell (percent.mt). As shown in Figure 1C, all samples included about 3000 genes. The sequencing counts for all samples were about 100,000. The percent.mt for all samples was about 20% because the retinas are highly energy-consuming tissues 12. The cells with nFeature  > 200 and percent.mt < 20% were selected for the subsequent analysis (Figure 1C).

Following data pre-processing, the sequencing data from 17510 cells in P12C retinas, 17132 cells in P12T retinas, 21618 cells in P17C retinas, and 19904 cells in P17T retinas were obtained. After integrating these cells from P12C, P17C, P12T, and P17T samples, we obtained a total of 76164 high-quality cell profiles. Graph-based clustering was carried out to group 76164 retinal cells according to gene expression profile. Finally, the clusters were annotated by comparing against the known marker genes of specific retinal cell types and visualized by the Uniform Manifold Approximation and Projection (UMAP). Based on the expression of the well-established retinal cell markers, we identified 10 different cell types, including photoreceptors, endothelial cells, amacrine cells, bipolar cells, pericytes, pigmented epithelial cells, microglial cells, Müller cells, erythroid cells, and retinal ganglion cells (Figure 1D).

### Identification of pericyte sub-population 2 vulnerable to pathological angiogenesis in OIR model

**Figure 2:**
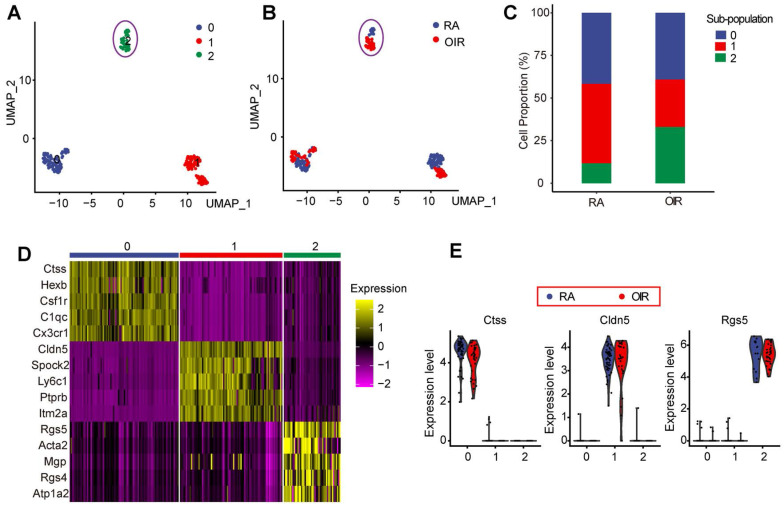
** Identification of pericyte sub-population 2 vulnerable to pathological angiogenesis in OIR model.** (A) Pericytes were color-coded by subpopulations and identified by an unsupervised graph-based algorithm, UMAP. (B) Identification of pericytes in OIR retinas and RA control retinas. Pericytes were color-coded by sample identity. (C) Bar graph showing the percentage of pericyte sub-populations in OIR retinas and RA control retinas. (D) Heatmap showing the expression of the top 5 high-abundance genes in pericyte sub-population 0, 1, and 2. (E) Violin plots showing the highest abundant gene in pericyte sub-population 0, 1, and 2 in OIR group and RA control group. Each dot represented one cell and the violin showed the distribution of probability density at each value.

Pericytes play critical roles in the process of pathological angiogenesis. Nonetheless, the transcriptomic and signaling heterogeneity of pericytes during pathological angiogenesis remains poorly understood 13. We thus filtered out pericytes using its markers and performed further analysis to identify the sub-populations of pericytes. A dimensionality reduction approach, UMAP, was employed to investigate the heterogeneity of pericytes during pathological angiogenesis. UMAP reduction analysis identified 3 transcriptionally distinct pericyte sub-populations (Figure 2A) and identified RA control pericytes and OIR pericytes (Figure 2B). To determine which pericyte sub-population was vulnerable to pathological angiogenesis in OIR model, we compared the proportions of various pericyte sub-populations in OIR retinas and control RA retinas at P17. The number of pericyte sub-population 2 in OIR retinas was significantly greater than that in RA control retinas. By contrast, the number of pericyte sub-population 0 or sub-population 1 in OIR retinas was smaller than that in the control RA retinas (Figure 2C). Moreover, there was nearly no obvious intersections for sub-population 2 between OIR group and RA group, suggesting that sub-population 2 in OIR group was significantly different from sub-population 2 in RA control group (Figure 2A-B). We thus speculate that pericyte sub-population 2 was potentially involved in the process of pathological angiogenesis.

The expression of the top 5 high-abundance genes in pericyte sub-population 0, 1, and 2 was listed in Figure 2D. The highest abundant gene in pericyte sub-population 0, 1, and 2 in OIR group and RA control group was shown in Figure 2E. In sub-population 0, Ctss, Hexb, Csf1r, C1qc, and Cx3cr1 are shown as important regulators of antigenic protein presentation, complement system, macrophage regulation, or leukocyte function, suggesting that pericyte sub-population 0 is mainly involved in vascular inflammatory responses. In sub-population 1, Cldn5, Spock2, Ly6c1, Ptprb, and Itm2a are known as the regulators of plasma membrane maintenance, tight junction component, or extracellular matrix formation, suggesting that pericyte sub-population 1 is mainly involved in the maintenance of these existing vessels. In sub-population 2, Rgs5, Acta2, Mgp, and Rgs4 are shown as the critical regulators of cell mobility, cell proliferation, and cell differentiation, suggesting that pericyte sub-population 2 is prone to be activated and is potentially involved in pathological angiogenesis.

### The potential function of pericyte sub-population 2

**Figure 3:**
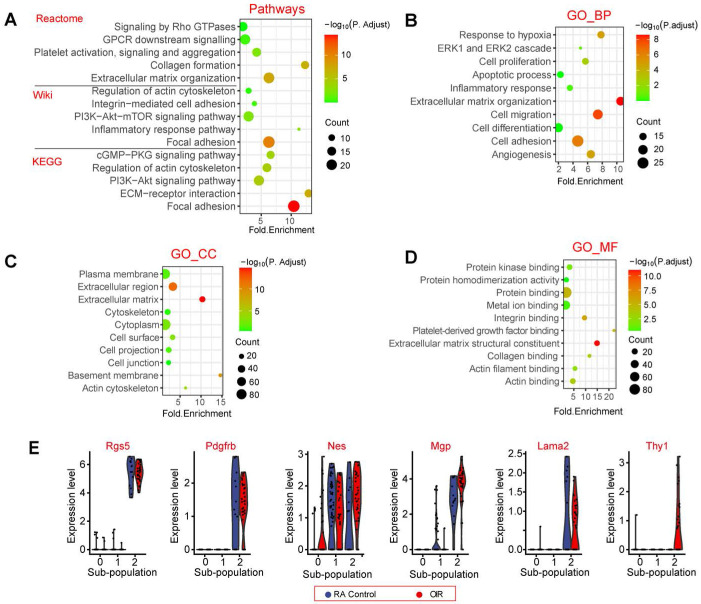
** The potential functions of pericyte sub-population 2.** (A) Dot plot showing the pathway enrichment results from three independent pathway databases, including Reactome, Wiki, and KEGG. The lists of the top 200 highly expressed genes in pericyte sub-population 2 were used as the inputs. (B - D) GO enrichment of the top 200 highly expressed genes in pericyte sub-population 2, including three ontologies: biological process (BP, B), cellular component (CC, C), and molecular function (MF, D). (E) Violin plots showing pericyte marker genes and the highly expressed genes in pericyte sub-population 2. Each dot represents one cell and the violin shows the distribution of probability density at each value.

We then carried out signaling pathway enrichment and Gene Ontology (GO) enrichment analysis using the top 200 highly expressed genes in pericyte sub-population 2. Pathway enrichment analysis revealed that the top 200 highly expressed genes in pericyte sub-population 2 were highly enriched in the following pathways, including focal adhesion, extracellular matrix organization, PI3K-Akt-mTOR signaling, ECM-receptor interaction, and collagen formation (Figure 3A). To further determine the biological functions of the top 200 highly expressed genes in pericyte sub-population 2, we conducted GO term enrichment analysis according to the three categories of biological process (BP), cellular component (CC), and molecular function (MF). These functional terms were largely linked with angiogenesis and extracellular matrix remodeling, such as cell adhesion, cell migration, and extracellular matrix organization, which may occur in the plasma membrane or extracellular matrix (Figure 3B-D). Some pericyte marker genes and highly expressed genes in pericyte sub-population 2 were listed in Figure 3E.

### Single-cell profile reveals the unique gene expression in pericyte sub-population 2

**Figure 4:**
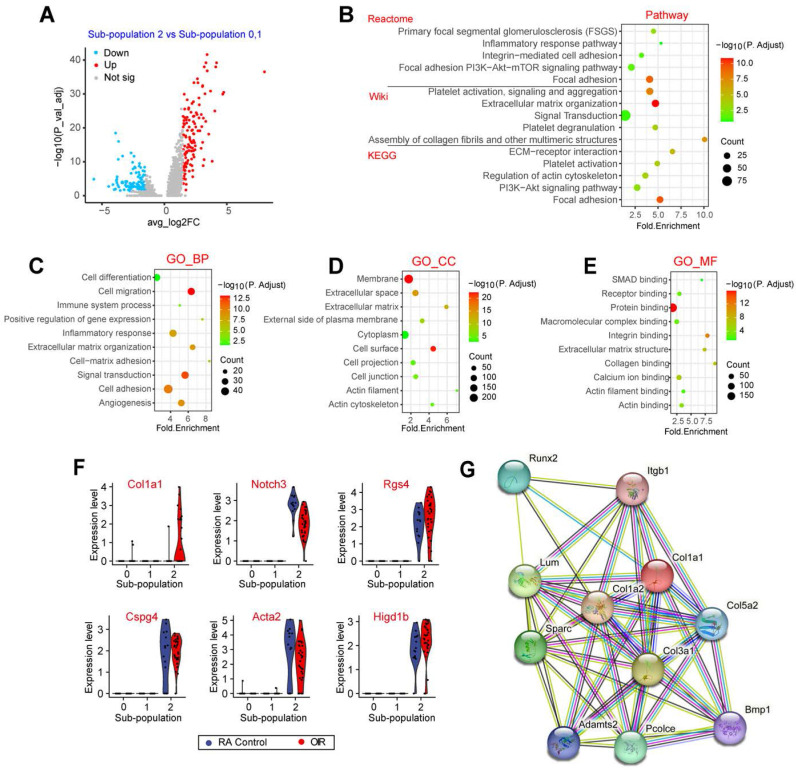
** Single-cell profile reveals the unique gene expression in pericyte sub-population 2.** (A) Volcano plot showing differentially expressed genes between sub-population 2 and sub-population 0, 1. (B) Dot plot showing the pathway enrichment results from three independent pathway databases. The lists of the differentially expressed genes between sub-population 2 and sub-population 0, 1 were used as the inputs for the analysis. (C-E) GO enrichment of the differentially expressed genes between sub-population 2 and sub-population 0, 1, including three ontologies of GO: biological process (BP, C), cellular component (CC, D), and molecular function (MF, E). (F) Violin plots showing the pericyte marker genes and the differentially expressed genes between sub-population 2 and sub-population 0, 1. Each dot represents one cell and the violin shows the distribution of probability density at each value. (G) The protein interaction network of Col1a1 was analyzed by the STRING network.

The above-mentioned evidence has revealed that pericyte sub-population 2 was tightly associated with the process of pathological angiogenesis. To investigate the transcriptomic heterogeneity of pericyte sub-population 2, we compared the expression profiles and the key marker genes of pericytes between sub-population 2 and other pericyte sub-populations (0 and 1). The results showed that 278 genes were significantly up-regulated in sub-population 2 and 168 genes were significantly down-regulated in sub-population 2 (Figure 4A). Pathway enrichment analysis revealed that these differentially expressed genes in pericyte sub-population 2 were greatly enriched in focal adhesion, signaling transduction, extracellular matrix organization, and ECM-receptor interaction (Figure 4B). GO term enrichment analysis further revealed that the differentially expressed genes between sub-population 2 and other pericyte sub-populations were largely linked to extracellular matrix remodeling, such as cell adhesion and ECM organization, which may occur at the membrane or extracellular matrix (Figure 4C-E). ECM is a series of structural and functional extracellular proteins, which is composed of a relaxed meshwork of collagens, elastin, and fibronectin. Focal adhesion is the physical action between cells and ECM. ECM remodeling plays important roles in multiple angiogenic processes including EC motility, proliferation, survival, and vessel formation 14. Notably, Type I collagen is the main ECM constituent, which is critical for EC proliferation and supporting EC survival 15. Some pericyte marker genes and extracellular matrix-related genes were listed in Figure 4F. Notably, Col1a1 was uniquely expressed at a high level in sub-population 2 but not in other sub-populations. We also mapped the Col1a1 onto STRING network (http://string.embl.de/) and constructed the interaction network with the confidence score greater than 0.893. The network was shown in Figure 4G. These genes had 45 interactions, which were much more than expected 12 edges with PPI (Protein-Protein Interaction) enrichment *P*-value smaller than 1.48e-13. Moreover, the interacting proteins of Col1a1 were tightly associated with ECM remodeling, such as Col1a2, Col3a1, Col5a2, and Bmp1 (Figure 4G).

### Col1a1 is increased in OIR retina and is highly expressed in pericytes

**Figure 5:**
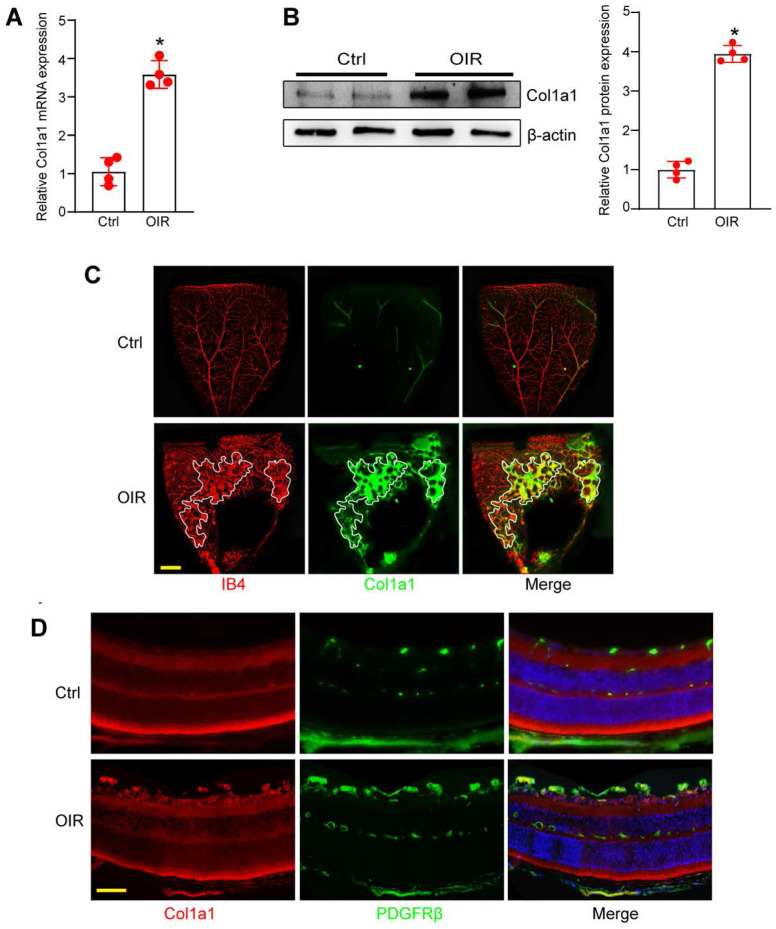
** Col1a1 is increased in OIR retina and is highly expressed in pericytes.** (A and B) qRT-PCR assays and western blot assays were conducted to compare Col1a1 expression between OIR retinas and RA control retinas at P17 (n = 4, *P < 0.05, Student's *t* test). (C and D) Immunofluorescence assays were conducted to detect Col1a1 expression in retinal flat-mounts and retinal cryosections. Meanwhile, IB4 or PDGFRβ was used to label retinal vessels or retinal pericytes. Scale bar: 100 μm.

Since Col1a1 is abundantly expressed in pericyte sub-population 2, we subsequently investigated its expression pattern and its potential role in angiogenesis. qRT-PCRs and western blots revealed that Col1a1 expression was markedly increased in the retinas of OIR mice at P17 compared with RA controls (Figure 5A-B). In retinal flat mounts, immunofluorescence assays revealed that Col1a1 was co-localized with retinal vascular marker, Isolectin B4 (IB4). Moreover, its expression was significantly increased in angiogenic regions (Figure 5C). In the retinal cryosections, immunofluorescence assays revealed the co-localization between Col1a1 and a pericyte marker, PDGFRβ (platelet derived growth factor receptor β), suggesting that Col1a1 was expressed in pericytes (Figure 5D). Collectively, the above-mentioned evidences indicate that Col1a1 is mainly expressed in pericytes and is potentially involved in angiogenesis.

### Col1a1 regulates pericyte biology *in vitro*

**Figure 6:**
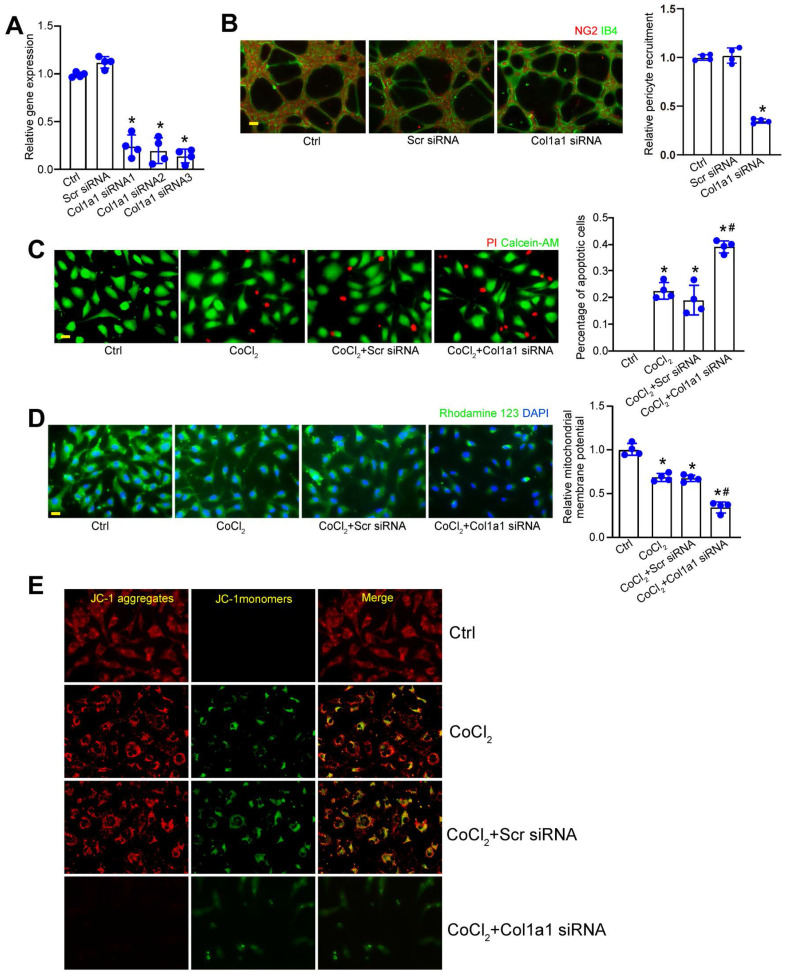
** Col1a1 regulates pericyte biology *in vitro*.** (A) Pericytes were transfected with scramble (Scr) siRNA, Col1a1 siRNA1-3, or left untreated for 24 h. qRT-PCRs were conducted to detect Col1a1 expression (n = 4). (B) Following the tranfection with Scr siRNA, Col1a1 siRNA3, or left untreated (Ctrl) for 24 h, pericytes were co-cultured with HRVECs for 6 h and then stained with NG2 (pericytes) and IB4 (HRVECs) to detect the recruitment of pericytes toward HRVECs (n = 4). Scale bar: 20 μm. (C-E) Pericytes were transfected with scramble (Scr) siRNA, Col1a1 siRNA1-3, or left untreated (Ctrl) for 24 h, and then exposed to CoCl_2_ for 48 h. The live and apoptotic pericytes were determined using Calcein-AM/PI staining. Green, live cells; red, dead, or dying cell. Scale bar: 20 μm (C, n = 4). Rhodamine 123 (D, n = 4) and JC-1 staining (E, n = 4) was performed to detect the change of mitochondrial membrane potentials. Rhodamine 123, green Red fluorescence; JC‐1 aggregates; green fluorescence, JC‐1 monomers; DAPI, blue. Scale bar: 20 µm. *P < 0.05 versus Ctrl group; ^#^*P* < 0.05 Scr siRNA group versus Col1a1 siRNA group.

We next determined whether Col1a1 silencing could affect pericyte biology *in vitro*. Three different Col1a1 siRNAs were designed for Col1a1 silencing (Table S1). Transfection of Col1a1 siRNA led to decreased levels of Col1a1 expression in pericytes. Notably, Col1a1 siRNA3 had the greatest inhibitory effects on Col1a1 expression (Figure 6A). The crosstalk between pericytes and endothelial cells have been implicated in pathological angiogenesis. Pericyte recruitment is critical for the process of pathological angiogenesis 16. Matrigel co-culture assays revealed that Col1a1 silencing in pericytes significantly decreased the number of human pericyte recruitment toward HRVECs (Figure 6B). Hypoxic stress is a pathological factor involved in pathological angiogenesis. We then investigated the effects of Col1a1 silencing on pericyte biology under hypoxic condition. Pericytes were exposed to CoCl_2_ to mimic hypoxic stress. Calcein‐AM/PI staining assays revealed that compared with CoCl_2_-treated group, Col1a1 silencing could aggravate CoCl_2_-induced pericyte apoptosis as shown by increased PI-positive cells (Figure 6C). Mitochondrial depolarization is tightly associated with cell apoptosis. We further conducted Rhodamine 123 staining and JC-1 staining to detect the change of mitochondrial depolarization in pericytes. Rhodamine 123 is a green dye that stains mitochondria in living cells in a membrane potential-dependent manner. JC-1 dye is an indicator of mitochondrial membrane potential and mitochondrial depolarization is indicated by a decrease in the red/green fluorescence intensity ratio. Compared with CoCl_2_-treated group, Col1a1 silencing further aggravated CoCl_2_-induced mitochondrial depolarization in pericytes (Figure 6D-E). Overall, these results suggest that Col1a1 is a critical regulator of pericyte biology *in vitro*.

### Col1a1 regulates retinal pericyte function and vascular integrity *in vivo*

**Figure 7:**
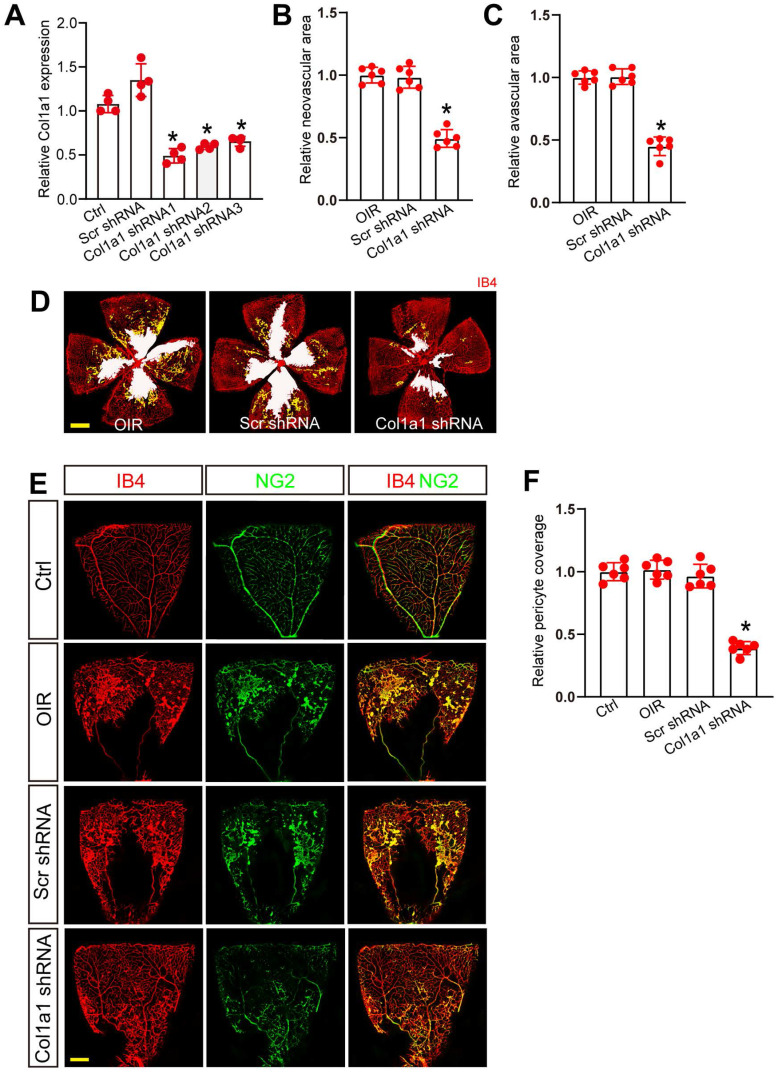
** Col1a1 regulates retinal pericyte function and vascular integrity *in vivo*.** (A) C57BL/6J mouse pups at the postnatal day (P)7 received an intravitreous injection of scrambled (Scr) shRNA or Col1a1 shRNA 1-3, or left untreated (Ctrl) for 1 week. The expression levels of Col1a1 were detected by qRT-PCRs (n = 4 mice per group). (B-D) C57BL/6J mouse pups received an intravitreous injection of Scr shRNA or Col1a1 shRNA1, or left untreated (Ctrl) for 1 week. Then, OIR models were established. The representative images of retinal flat-mounts were stained with IB4. Scale bar, 100 μm. Yellow staining indicated neovascular area; white staining indicated avascular area. The quantification results of neovascular area and avascular area were also shown (n = 6 mice per group). (E and F) C57BL/6J mouse pups received an intravitreous injection of Scr shRNA or Col1a1 shRNA1, or left untreated for 1 week. Then, OIR models were established. The room air-treated mice were taken as the Control (Ctrl) group. Pericyte coverage was quantified by staining retinal flat-mounts with IB4 and NG2. To visualize a whole leaf of retinal vessel, tile scanning was used whereby multiple overlapping (10%-20% overlap) images were captured by a ×10 lens with identical gain setting. The composite images were generated by arraying the individual images in Adobe Photoshop. Scale bar: 100 μm. The quantification results of pericyte coverage in retinal vessels were also shown (n = 6 mice per group). All significant difference was determined by one-way ANOVA assay. *P < 0.05.

We next determined the role of Col1a1 in retinal pericyte function and vascular integrity *in vivo*. Three short hairpin RNAs (shRNAs) targeting Col1a1 were designed and inserted into the virus vector to obtain Col1a1 shRNAs (Table S1). We then evaluated the silencing efficiency by qRT-PCR assays. The results showed that Col1a1 shRNA1 had the greatest silencing efficiency on Col1a1 expression in the retinas (Figure 7A). We thus selected Col1a1 shRNA1 to conduct the subsequent experiments.

We built ROP model to investigate the role of Col1a1 in retinal vascular dysfunction. Compared with the OIR group, injection of Col1a1 shRNA1 but not Scr shRNA significantly reduced the size of neovascular area and avascular area in OIR retinas at P17 (Figure 7B-D). Meanwhile, IB4 and NG2 immunofluorescence staining was conducted to determine the effects of Col1a1 silencing on pericyte coverage in retinal vessels. Compared with Ctrl group, pericyte/EC ratio in retinal vessels did not change in the OIR group or OIR plus Scr shRNA group. However, injection of Col1a1 shRNA1 led to reduced pericyte/EC ratio in retinal vessels, suggesting that Col1a1 silencing led to a marked reduction of pericyte coverage in retinal vessels (Figure 7E-F). Taken together, these data suggest that Col1a1 regulates retinal pericyte function and vascular integrity *in vivo*.

### Col1a1 regulates pericyte-myofibroblast transition and endothelial-mesenchymal transition

**Figure 8:**
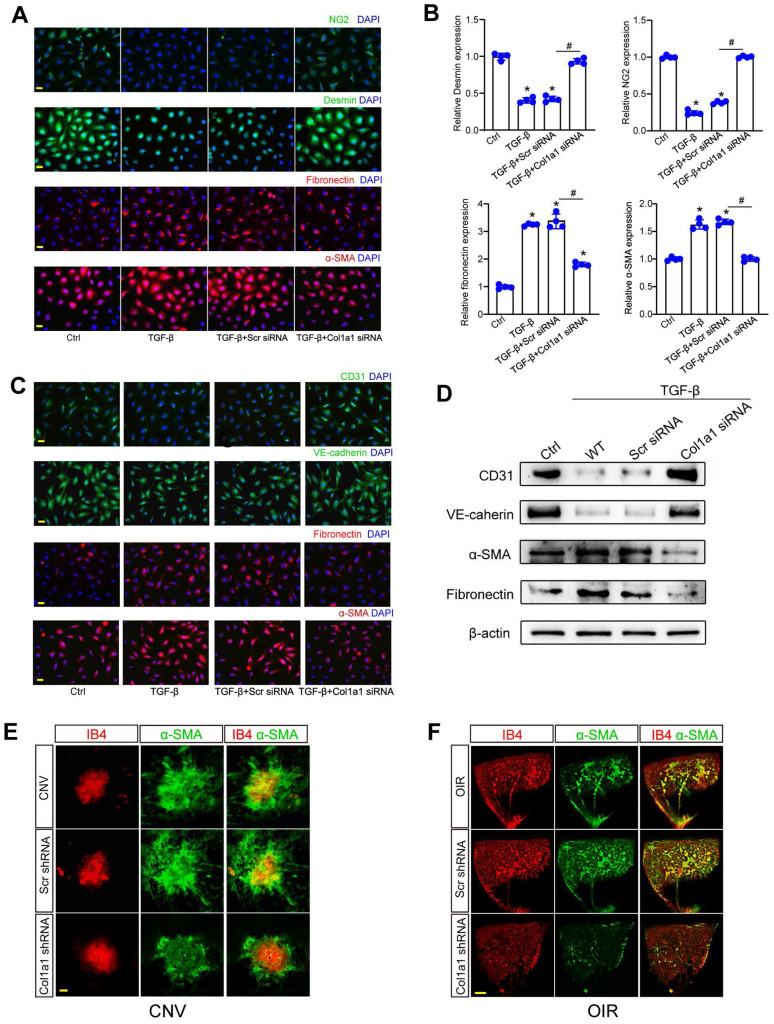
** Col1a1 regulates pericyte-myofibroblast transition and endothelial-mesenchymal transition.** (A and B) Pericytes were transfected with Scr siRNA, Col1a1 siRNA, or left untreated (Ctrl) for 48 h. IF assays (A) and qRT-PCR assays (B) were conducted to detect the expression of Desmin, NG2, fibronectin, and α-SMA to estimate the degree of pericyte-myofibroblast transition (n = 4; *P < 0.05 versus Ctrl; ^#^*P* < 0.05 TGF-β+Scr siRNA versus TGF-β+Col1a1 siRNA; One-way ANOVA). (C and D) The noncontact co-culture models, including ECs and untreated pericytes or pericytes transfected with Scr siRNA or Col1a1 siRNA for 12 h were built. Then, ECs were exposed to TGF-β1 (10 ng/ml) for 48 h. IF assays (C) and western blots (D) were conducted to detect the expression of CD31, VE-cadherin, fibronectin, and α-SMA to estimate the degree of endothelial-mesenchymal transition (n = 4). Scale bar: 20 μm. (E) After laser treatment, C57BL/6J mice received an intravitreous injection of Scr shRNA or Col1a1 shRNA1, or left untreated (Ctrl) for 1-week. Then, laser-induced CNV model was built. The representative images of RPE/choroidal flat mounts stained by IB4 and α-SMA were shown. n = 6; Scale bar: 100 μm. (F) C57BL/6J mouse pups at P7 received an intravitreous injection of Scr shRNA, Col1a1 shRNA1, or left untreated (Ctrl). Then, OIR models were established. Representative images of retinal flat-mounts stained by IB4 and α-SMA were shown. n = 6; Scale bar: 100 μm.

Since Col1a1-positive pericytes are tightly associated with pathological angiogenesis, we further investigated the role of Col1a1 in pericyte biology. Under pathological conditions, pericytes are activated, detached from vascular walls, and transformed into myofibroblasts. Pericyte-myofibroblast transition (PMT) can activate subsequent capillary inflammation and fibrosis 17, 18. In pericytes, Col1a1 silencing could suppress the expression of Desmin and NG2 and potentiate the expression of fibronectin and a-SMA (Figure 8A-B). Crosstalk between pericytes and ECs was also involved in the process of pathological angiogenesis 19. Cell co-culture assays revealed that Col1a1 silencing in pericytes retarded the progression of endothelial-mesenchymal transition (EndoMT). Col1a1 silencing in pericytes could reverse TGF-β1-induced up-regulation of extracellular matrix markers (fibronectin and α-SMA) and TGF-β1-induced reduction of endothelial markers (CD31 and VE-cadherin) in ECs (Figure 8C-D), suggesting that Col1a1 silencing in pericytes has an indirect effect on EC biology.

α-SMA is an important marker for capillary matrix remodeling during pathological angiogenesis, which is abundantly expressed in pericytes and myofibroblasts 18. We used laser-induced CNV model and OIR model to investigate the role of Col1a1 silencing on α-SMA expression. Compared with CNV group (Figure 8E) or OIR group (Figure 8F), Col1a1 silencing did not affect the levels of IB4 expression but led to a marked reduction of α-SMA expression, suggesting that Col1a1 silencing retards capillary matrix remodeling, which may ultimately retard the progression of pathological angiogenesis.

### Clinical relevance of Col1a1 dysregulation in retinal vascular diseases

**Figure 9:**
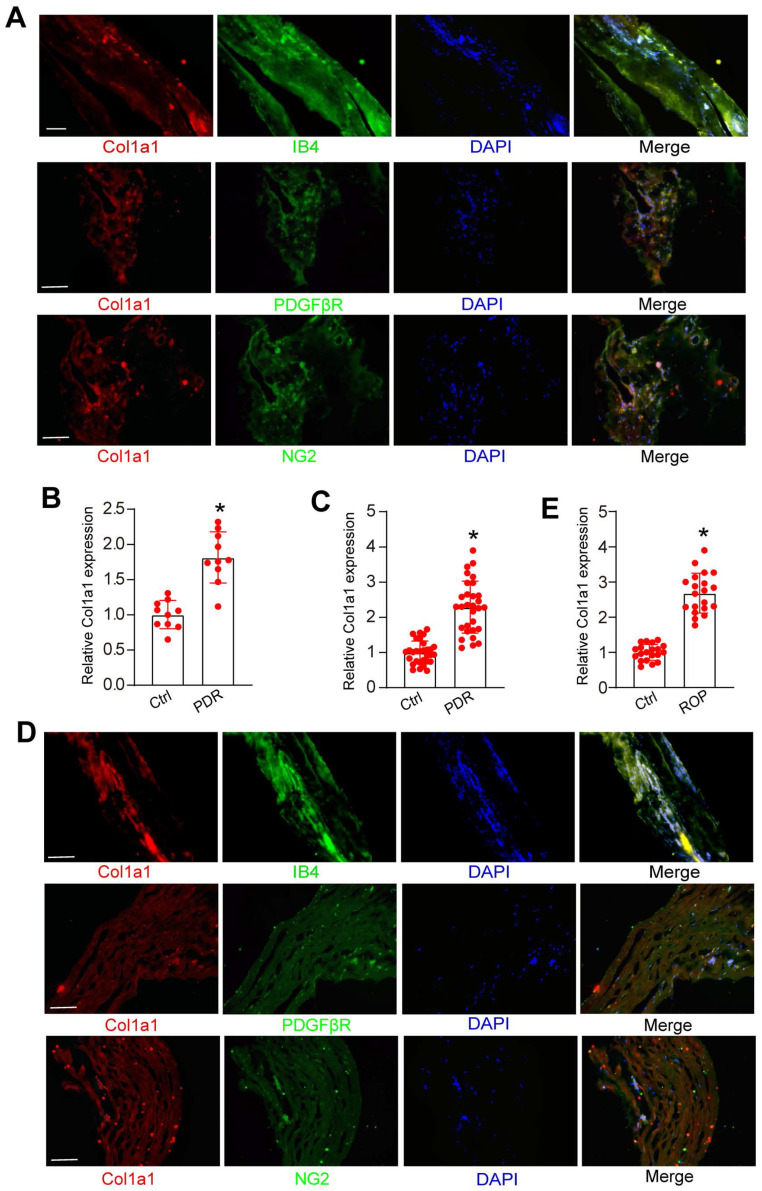
** Clinical relevance of Col1a1 dysregulation in retinal vascular diseases.** (A) Immunofluorescence assays were conducted to detect the expression distribution of Col1a1 in neovascular fractions and pericytes (PDGFβR and NG2) in the proliferative membranes of PDR patient. Scale bar: 100 μm. (B) The proteins were isolated from the proliferative membranes of PDR patients and the epiretinal membranes of non-diabetic patients (Ctrl). ELISA assays were conducted to compare the expression difference of Col1a1 (n = 10; *P < 0.05 versus Ctrl; One-way ANOVA). (C) AH samples were collected from the patients with PDR and age-matched cataract patients without any ocular diseases (Ctrl). ELISA assays were conducted to compare the expression difference of Col1a1 (n = 30; *P < 0.05 versus Ctrl; One-way ANOVA). (D) Immunofluorescence assays were conducted to detect the expression distribution of Col1a1 in neovascular fractions and pericytes (PDGFβR and NG2) in the proliferative membranes of ROP patients. Scale bar: 100 μm. (E) AH samples were collected from the patients with ROP and age-matched cataract patients (Ctrl). ELISA assays were conducted to compare the expression difference of Col1a1 (n = 10; *P < 0.05 versus Ctrl; One-way ANOVA).

We further investigated whether Col1a1 dysregulation occurred in retinal vascular diseases. PDR is an advanced-stage proliferative diabetic retinopathy characterized by neovascularization or preretinal/vitreous haemorrhages. Immunofluorescence assays revealed that Col1a1 was abundantly expressed in the fibrovascular membranes of PDR patients and was co-localized with neovascular fractions (IB4 staining, Figure 9A). Moreover, Col1a1 was co-localized with pericyte markers (PDGFRβ and NG2 staining, Figure 9A). We then detected the levels of Col1a1 expression in the fibrovascular membranes of PDR patients and the epiretinal membranes of non-diabetic patients. ELISA assays revealed that the levels of Col1a1 expression were significantly up-regulated in the fibrovascular membranes of PDR patients (Figure 9B). We further collected the aqueous humor (AH) samples from PDR patients and age-matched patients without ocular neovascular disease. ELISA assays revealed that compared with the control group, higher levels of Col1a1 were detected in the AH samples of PDR patients (Figure 9C).

The afore-mentioned result revealed that Col1a1 expression obviously increased in OIR mouse retinas. We speculated that Col1a1 was also dysregulated in the clinical samples of ROP patients. Immunofluorescence assays revealed that Col1a1 was co-stained with IB4 staining in the idiopathic epiretinal membranes of ROP patients. Col1a1 was co-localized with pericyte markers in the idiopathic epiretinal membranes of ROP patients (PDGFRβ and NG2 staining, Figure 9D). ELISA assays showed that the levels of Col1a1 expression were significantly up-regulated in AH samples of ROP patients (Figure 9E). Together, these results suggest that Col1a1 dysregulation is involved in the pathogenesis of retinal vascular diseases.

## Discussion

Reduced blood flow and impaired capillary function is a hallmark of neovascular diseases, including ocular vascular diseases and cancers 20. Although anti-angiogenic molecules have been discovered that can suppress pathological angiogenesis, such as anti-VEGF blockers. However, anti-VEGF blockers not only inhibit pathological angiogenesis but also damage the healthy vessels, resulting in toxic side effects. Moreover, some patients are refractory or acquire the resistance to anti-VEGF blockers 21, 22. Thus, it is still required to further investigate the angiogenic mechanism to identify novel anti-angiogenic molecules. Herein, we performed single-cell RNA sequencing to unravel the contribution of pericyte sub-populations to capillary dysfunction.

Capillaries are mainly composed of pericytes and endothelial cells (ECs). Pericytes are vascular mural cells embedded in the basement membranes of capillaries 23. For a long time, their existence and roles have been neglected. However, increasing studies have shown that pericytes are key players during capillary dysfunction, which can regulate vessel development and remodeling 24, 25. However, the molecular profile and heterogeneity of pericytes are poorly understood. Herein, we utilized high-throughput scRNA-seq to investigate the heterogeneity of pericytes associated with pathological angiogenesis. The results show that retinal pericytes are highly heterogeneous and are mainly composed of 3 different subpopulations, including an activated pericyte subpopulation with pro-angiogenic capacity, a pericyte subpopulation with inflammatory response, and a resting pericyte subpopulation with homeostatic function. Notably, we detected a marked change of pericyte sub-population 2, which is spatially localized at retinal neovascularization (RNV) regions and is characterized by the abundant expression of Col1a1, indicating their involvement in pathological angiogenesis.

Col1a1 encodes the major component of type I collagen, which is shown as the collagen in most connective tissues 26. Type I collagen has been found in most connective tissue and embryonic tissue. It is a key structural component of extracellular matrix. Type I collagen comprises of two chains of collagen type I alpha 1 (Col1a1) and one chain of collagen type I alpha 2 (Col1a2) 27. Notably, the expression of Col1a1 directly correlates with the extent of fibrosis in the animal model. Abnormal expression of Col1a1 and Col1a2 has been reported in several cancers and other fibrotic diseases 28, 29. Mutations in Col1a1 gene have also been reported in several human diseases, such as myopia, type I osteogenesis imperfecta, Ehlers-Danlos and Marfan syndromes, and osteoporosis 30, 31. In this study, we found increased Col1a1 expression during pathological angiogenesis both at mRNA level and protein level. Col1a1 silencing could suppress ocular angiogenesis and suppress pericyte-myofibroblast transition during capillary remodeling, demonstrating a therapeutic role in the treatment of pathological angiogenesis and fibrotic responses.

Pericytes and perivascular fibroblasts have recently been identified as the source of scar-producing myofibroblasts following pathological angiogenesis 32. Pericyte-myofibroblast transition is known as an important pathological process involving fibrotic diseases. This process is characterized by the detachment of pericytes from endothelial walls and undergoes the transition into myofibroblasts 33. The new fibroblasts could activate inflammatory macrophages and cause capillary fibrosis 34. In this study, we reveal that Col1a1 is linked to mediate pericyte proliferation, migration as well as differentiation. Col1a1 silencing could reduce pericyte proliferation and decrease the number of pericyte recruitment toward endothelial cells. Moreover, Col1a1 silencing could exert protective effects on retinal vascular integrity and suppress pericyte-myofibroblast transition. Together, these evidences also suggest that Col1a1 silencing may embody the potential implications for the treatment of pathological angiogenesis and related fibrotic responses.

Pericytes are perivascular cells attached to the abluminal surface of capillaries. The crosstalk between pericytes and endothelial cells can concomitantly dictate capillary degeneration 35. Under the physiological condition, pericytes can maintain capillary quiescence and capillary integrity. Under the pathological condition, pericytes are activated and detach from vascular walls 36. Meanwhile, pericyte degeneration can lead to vulnerable capillaries, which are prone to instability, pathological angiogenesis, and ultimately rarefaction 37, 38. In this study, EC-pericyte co-culture assays reveal that Col1a1 silencing in pericytes significantly retards the development of endothelial-mesenchymal transition (EndoMT). ECs were treated with TGF-β1 to build EndoMT model *in vitro*. Col1a1 silencing in pericytes could reverse TGF-β1-induced up-regulation of fibronectin and α-SMA and reverse TGF-β1-induced down-regulation of CD31 and VE-cadherin in ECs. In laser-induced CNV model and OIR model, we further reveal that Col1a1 silencing causes a marked reduction of α-SMA and retard capillary extracellular matrix remodeling.

Single-cell RNA sequencing (scRNA-seq) has become a powerful and efficient tool to define cellular heterogeneity in disease condition 39, 40. In this study, we employed scRNA-seq method combined with genetic perturbation, *in vitro*, and *in vivo* functional analysis to comprehensively profile pericyte sub-populations in pathological angiogenesis. In OIR murine model, we unravel the spatial location of pericyte sub-population 2 adjacent to pathological neovascularization tufts. Moreover, we elucidate the role of the marker gene of pericyte sub-population 2, Col1a1, in pathological angiogenesis. Collectively, this study defines the contribution of pericyte heterogeneity to pathological angiogenesis at the single-cell level.

## Materials and methods

### Animal experiment

All experiments were conducted according to the guidelines of the Association for Research in Vision and Ophthalmology (ARVO) Statement for the Use of Animals in Ophthalmic and Vision Research and approved by the Institutional Animal Care and Use Committee of the authors' institute. C57BL/6J mice were purchased from Nanjing Qinglongshan Experimental Animal Center (Nanjing, China). They were bred in an air-conditioned room with a 12 h light-dark cycle and fed with standard laboratory chow and allowed free access to water.

### Oxygen-induced retinopathy (OIR) model

OIR model was induced in C57BL/6J mice on the postnatal day 7 (P7). At P7, the pups with their nursing mothers were placed in 75% oxygen for 5 days. At P12, they were removed from oxygen and returned to normoxia until P17. The age-matched control animals were reared simultaneously in room air 41.

### Single-cell library preparation and sequencing

The eyes were enucleated from OIR mice and room air (RA) control mice. The retinas were dissected in cold 1×PBS, minced into small pieces on ice, and enzymatically digested with the papain solution (For 1 ml: 700 μL reagent grade water, 100 μL of freshly prepared 50 mM L-Cysteine (Sigma), 100 μL of 10 mM EDTA, 10 μL of 60 mM 2-mercaptoethanol (Sigma), and papain added to 1 mg/ml (Worthington)) for 30 min at 37°C. Next, the single-cell solution was sieved via a 70-μM cell strainer (Corning) and red blood cells were removed. Cell number was counted and cell viability was determined with the BD Rhapsody scRNA-seq platform (BD Biosciences).

The isolated single-cells were processed with the BD Rhapsody scRNA-seq platform (BD Biosciences) 42, 43. The cells from all samples were labeled at room temperature for 20 min and washed by the centrifugation at 500 *g* for 5 min. Single-cell isolation in microwells with subsequent cell lysis and capture of the poly-adenylated mRNAs with the barcoded, magnetic capture-beads was performed according to the manufacturer's instructions. The beads were magnetically retrieved and pooled into a tube before reverse transcription. Then, the unique molecular identifiers (UMIs) were added to cDNA molecules during cDNA synthesis. The whole transcriptome amplification and the sample-tag sequencing libraries were generated based on the BD Rhapsody single-cell whole-transcriptome amplification workflow. The quantity and quality of sequencing libraries was detected using the Qubit dsDNA HS Assay Kit and the Agilent 4200 TapeStation system. The libraries were sequenced by the NovaSeq 6000 (Illumina) platform.

### Single-cell sequencing data analysis

Sample demultiplexing, read alignment to NCBI reference genome, quantification and initial quality control (QC) of microfluidics-based sequencing dataset was analyzed using the Seurat R package version 3.15.1 44. The sequencing results were presented in the format of expression matrix. In each sample, the cells with fewer than 200 detected genes and/or larger than 20% mitochondrial genes were excluded. Identification of cell clusters on UMAP (uniform manifold approximation and projection) were performed. Single R and the known retinal marker genes were used to annotate cell type. Differentially expressed genes of each cluster were identified using the FindAllMarkers function of Seurat. The enriched GO terms and KEGG pathways were identified using the online tool DAVID: https://david.ncifcrf.gov/.

### Cell culture and transfection

Human retinal vascular endothelial cells (HRVECs) were obtained from ATCC (American Type Culture Collection, USA). Human retinal pericytes were purchased from Cell Systems (Kirkland, WA). HRVECs were cultured in Dulbecco's modified Eagle's medium (DMEM) containing 10% fetal bovine serum (FBS, 10099141C, Gibco, USA), 1% antibiotic-antimycotic solution (penicillin/streptomycin, Gibco, USA) at 37°C and 5% CO_2_ in a humidified incubator. Pericytes were maintained in DMEM supplemented with 10% FBS, 1% antibiotic-antimycotic solution at 37°C in 5% CO_2_ incubator. Three Col1a1 siRNAs (Col1a1 siRNA 1-3) and negative control siRNA was purchased from Guangzhou RiboBio Co., Ltd. They were transfected at 70-90% confluence using Lipofectamine 3000 (Invitrogen, Grand Island, NY, USA). The efficiency of gene silencing was assessed by qRT-PCR assays at 48 h following siRNA transfection.

### Intravitreal injection

In OIR models, the pups received an intravitreal injection at P7 before they were exposed to 75% oxygen. Each eye was intravitreally injected with either 1 μl of virus (3×10^8^ TU/mL) containing Col1a1 shRNA or scrambled shRNA. Under a dissecting microscope, a fine glass micropipette connected to a 10 μL Hamilton glass syringe (Hamilton Co.,Reno, NV) was inserted through the incision in the cornea and slid between the iris and the lens into the posterior chamber of the eye. For C57BL/6 mice (8-week old), intravitreal injection was performed after they were anesthetized with a mixture of ketamine hydrochloride (50 mg/kg) and xylazine (10 mg/kg). Meanwhile, a topical anesthesia was applied over the injection site using the oxybuprocaine hydrochloride. Then, 2 μl of virus containing Col1a1 shRNA or scrambled shRNA was delivered into their vitreous. The eyes with len injury or vitreous hemorrhage after the injection were excluded. The injection site was compressed to avoid the reflux on the removal of the needle. Topical antibiotic ointment was administered four times daily for 3 days.

### Quantification of pericyte coverage

Retinal whole-mount was used for detecting pericyte coverage by immunofluorescence staining 2, 13. The eyes were enucleated and fixed in 4% PFA (Biosharp Biotechnology, BL539A) for 30 min at room temperature and then transferred to cold 1 × PBS on ice for 10 min. The neural retina and choroid/RPE were dissected and placed in cold formaldehyde. Next, the retinas were blocked with 5% BSA for 30 min, stained with NG2 (1:100, Abcam, ab50009) overnight at 4°C, and stained with the Alexa Fluor 594 goat anti-mouse IgG (1:500, Invitrogen, A11005) for 3 h at room temperature. The retinas were finally stained with Isolectin B4 (IB4, 1:100, MilliporeSigma, L2895) for 2 h at room temperature to label retinal vessels. Pericyte coverage was quantified using Image J software.

### Patients used for proliferative membrane study

This study was carried out according to the tenets of the Declaration of Helsinki and adhered to the ARVO statement on human subjects. This study was also approved by the Institutional Review Board of the authors' institute. Informed consent was obtained from all patients and controls. This study enrolled the patients with proliferative diabetic retinopathy (PDR) as the case group and the non-diabetic patients with epiretinal membranes as the control group. All patients were 50-68 years old and received pars plana vitrectomy with membrane peeling. The patients with the following conditions were excluded: a history of ocular trauma, intraocular surgery, and ocular inflammatory diseases. All membranes were fixed in 4% PFA for 24 h and then transferred to a 30% sucrose solution for 5 h. The membranes were embedded in OCT. Tissue sections (10-μm) were thawed, washed with PBS, and blocked with PBS containing 5% BSA at 37 ℃. The sections were incubated with Col1a1, IB4, PDGFRβ or NG2 antibody at 4°C for 24 h. After washing, the sections were incubated with an Alexa Fluor 594-conjugated or Alexa Fluor 488-conjugated secondary antibody for 2 h at room temperature. Finally, the sections were washed with PBST and stained with DAPI to label nuclei.

### Patients used for aqueous humor (AH) study

AH sample was collected from the patients with PDR, retinopathy of prematurity, and age-matched cataract patients. Approximately 30-50 μl of AH sample was collected from each patient using a 30-gauge needle inserted through the peripheral cornea. The needle did not touch any iris or lens tissue during the sample collection. AH sample was collected under the surgical microscope before cataract surgery and anti-VEGF intravitreal injection. AH samples were immediately transferred into the sterile tubes and centrifuged at 12,000 g for 5 min. The expression levels of Col1a1 in AH samples were detected using the commercial ELISA kits.

### Statistical analysis

All data were expressed as mean±SD. The statistical analysis was performed using GraphPad Prism 8. The significant differences were determined by 2-tailed Student's *t* test (2-group comparisons) or one-way analysis of variance (ANOVA) with Tukey's post hoc test (multiple group comparisons) where appropriate. *P*<0.05 was considered statistically significant.

## Supplementary Material

Supplementary materials and methods, table.
