# Supplementary material for: Single-cell RNA sequencing reveals a unique pericyte type associated with capillary dysfunction
Source: Theranostics. 2023 Apr 23;13(8):2515–30. doi: 10.7150/thno.83532 (PMC10196835; doi:10.7150/thno.83532)
Supplement: Supplementary file 1 — Supplementary materials and methods, table. [file thnov13p2515s1.pdf]

Supplemental data

Supplemental materials and methods

Laser-induced choroidal neovascularization model

Eight-week-old male C57BL/6J mice were anesthetized by intraperitoneal injection of ketamine hydrochloride (50 mg/kg) and xylazine (10 mg/kg). Their pupils were dilated with 0.5% tropicamide eye drops. Four laser burns surrounding the optic disc were induced by the Supra Scan 577 nm continuous laser mode (Quantel Medical, France). The induction was conducted in the following settings: a 532-nm laser, 120 mW power, 50 um spot size and 100 ms duration. The breakage of Bruch's membrane was confirmed by a white central bubble formation at the injured area. One week after the rupture of Bruch membrane, the enucleated eyes were fixed in 4% paraformaldehyde (PFA) for 30 min. After removing the cornea, lens, vitreous, and retina, the posterior eyecups were blocked with PBS containing 5% BSA and 1% Triton X-100 at 37°C and followed by staining with the primary antibody at 4°C overnight. The choroidal flat mounts were finally stained with IB4 and the secondary fluorescence antibody (1:200, Molecular Probes) for 2 h at room temperature. The inclusion criteria were the formation of a bubble following laser burn and the absence of subretinal hemorrhage. Lesions violating the criteria were excluded.

Mitochondrial depolarization assay

Following the required treatment, pericytes were washed with PBS buffer and stained with JC-1 (5 μg/mL, Beyotime Biotechnology, Shanghai, China) at 37°C for 30

min to detect the change of mitochondrial membrane potential. Then, these cells were washed with PBS three times and observed under a fluorescence microscope (excitation wavelength: 475 nm; green emission: 530 nm; red emission: 590 nm). In the control cells, an intact $\Delta\psi m$ allows the JC-1 dye in a delocalized positive charge, exhibiting a red fluorescence. In the apoptotic cells, $\Delta\psi m$ collapse allows JC-1 dye in the cytoplasm, exhibiting a green fluorescence.

Rhodamine 123 staining

After the required treatment, pericytes were harvested and suspended in PBS. They were stained with Rhodamine 123 (R8030, Solarbio, China) for 1 h in the dark at 37°C. The cells were then washed three times with PBS and resuspended. The change of mitochondrial membrane potential was observed under a fluorescence microscope at the excitation and emission wavelengths of 488 nm and 594 nm.

Calcein-AM and propidium iodide (PI) double staining

Pericytes were seeded in 24-well plates overnight in DMEM with 10% FBS. After the required treatment, they were stained with PI and Calcein-AM (Beyotime Biotechnology, Shanghai, China) dyes at a 1:1 ratio at 37 °C for 20 min. Finally, the signaling was observed under a fluorescence microscope. The viable cells were observed at 490 nm excitation filter, while the dead cells were observed at 545 nm excitation filter.

Matrigel co-culture assay

The Matrigel was added to the 24-well plate and incubated at 37 °C for 30 min to allow Matrigel matrices to polymerize. Meanwhile, HRVECs and pericytes were

seeded onto the 24-well plate pre-coated with Matrigel and covered with DMEM plus FBS medium. After 6 h culture, these cells were fixed in 4% PFA for 15 min, stained with Isolectin B4 (IB4) (1:50, Invitrogen, I21413) to label endothelial cells, and then stained with anti-NG2 (1:200, Santa Cruz Biotechnology, 53389) to label pericytes. If the two colors merged together, that means that pericytes were recruited to endothelial cells.

Western blot

Cells or tissues were lysed in the RIPA buffer containing protease and phosphatase inhibitors (Sigma-Aldrich). The lysates were resolved by SDS-PAGE and the proteins were transferred to Immobilon-P PVDF (Millipore) and blocked with 5% BSA. The membranes were subjected to western blot using the primary antibody overnight at 4°C. The membranes were rinsed with 1× Tris-Buffered Saline Tween-20 (TBST) solution, incubated with the appropriate peroxidase-conjugated secondary antibody for 2 h, and washed with 1× TBST solution. The membranes were detected with an enhanced chemiluminescence system (Tiangen Biotech Co.) and relative intensities of protein bands were analyzed by Image J software (U.S. National Institutes of Health, Bethesda, MD, USA).

RNA isolation and quantitative reverse transcription PCR (qRT-PCR)

Total RNAs were extracted from pericytes or retinal tissues using the FastPure Cell/Tissue Total RNA Isolation Kit (Vazyme Biotech, China) according to the manufacturer's instructions. Reverse transcription was conducted using the HiScript III RT SuperMix for qPCR (Vazyme Biotech, China). To determine the expression levels

of target genes, qPCR assays were performed using the cDNA templates on a PikoReal Real-Time PCR System (Thermo Scientific) as shown below: initial denaturation at 95 ˚C for 3 min, followed by 42 cycles at 95 ˚C for 10 sec, 56 ˚C for 30 sec and 72 ˚C for 30 sec, with final extension at 72 ˚C for 60 sec. Each 20-μL reaction mixture contained 10 μL of 2 × SYBR Premix Ex Taq™, 2 μL of diluted cDNA, 2 μL of each primer (2 μM), 0.4 μL of ROX Reference Dye (50×) and 3.6 μL of double distilled water. β-actin was used as the internal control. The specific primers for the detected genes were listed in Table S1

Immunofluorescence staining

For cell immunofluorescence, pericytes were fixed in 4% PFA for 30 min, rinsed with PBS, and incubated for 30 min in blocking solution (5% BSA, 0.1% Triton X-100). Then, they were incubated with the primary antibody overnight at 4°C. After washing with phosphate buffered solution (PBST), they were stained with the corresponding fluorescent secondary antibody for 2 h in the dark at room temperature. The nuclei were counterstained with 4’, 6-diamidino-2-phenylindole (DAPI) (1:3000, Beyotime Biotechnology).

For retinal immunofluorescence, the eyes were fixed in 4% PFA overnight, dehydrated in 30% sucrose solution, embedded in optimal cutting temperature compound (OCT), and prepared for 10 μm cross-section. After permeabilization with 1% Triton X-100 and blocking with 5% BSA, the slices were incubated with the primary antibody overnight at 4 ℃. After washing, the slices were stained with the secondary antibody for 2 h in the dark at room temperature. DAPI was used to label

cell nuclei.

COL1A1 shRNA lentiviral construction

Col1a1 shRNAs were designed and produced by Shanghai Genechem Co., Ltd and ligated into pGV112 (Hu6-MCS-CMV-puromycin) (GeneChem, Shanghai, China). Three different Col1a1 RNAi target sequences show in Table S1. A scrambled shRNA with no homology within the mouse and rat genome was used as the control. Viral vector particles were then produced with a final titer of 3×10^8 TU/mL. The package and amplification of virus vector was conducted in HEK293T cells.

Table S1: PCR primer sequence, siRNA sequence, and shRNA target sequence

**Primer sequence**	
COL1A1	
Forward sequence	5’-GAGGGCCAAGACGAAGACATC-3’
Reverse sequence	5’-CAGATCACGTCATCGCACAAC-3’
Col1a1	
Forward	5’-GTGGAAACCCGAGGTATGCT-3’
Reverse	5’-GGTCCCTCGACTCCTACATCT-3’
β-ACTIN	
Forward	5’-TTGTTACAGGAAGTCCCTTGCC-3’
Reverse	5’-ATGCTATCACCTCCCCTGTGTG-3’
β-actin	
Forward	5’- GGCTGTATTCCCCTCCATCG -3’
Reverse	5’- CCAGTTGGTAACAATGCCATGT -3’
**siRNA sequence**	
Col1a1 siRNA1	5’-GCAAGACAGTGATTGAATA-3′
Col1a1 siRNA2	5’-GCAAGGTGTTGTGCGATGA-3′
Col1a1 siRNA3	5’-CCAGCTGTCTTATGGCTAT-3′
**shRNA target sequence**	
Col1a1 shRNA1	5’-CCTGGTGATACTGGTGTTAAA-3’
Col1a1 shRNA2	5’-GCCTTGGAGGAAACTTTGCTT-3’
Col1a1 shRNA3	5’-CCGAGGTATGCTTGATCTGTA-3’
